# Leveraging Lessons Learned from Yellow Fever and Polio Immunization Campaigns during COVID-19 Pandemic, Ghana, 2021

**DOI:** 10.3201/eid2813.221044

**Published:** 2022-12

**Authors:** Kwame Amponsa-Achiano, Joseph Asamoah Frimpong, Danielle Barradas, Delia Akosua Bandoh, Ernest Kenu

**Affiliations:** Expanded Program on Immunization, Ghana Health Service, Accra, Ghana (K. Amponsa-Achiano);; Ghana Field Epidemiology and Laboratory Training Programme, University of Ghana, Accra (J.A. Frimpong, D.A. Bandoh, E. Kenu);; US Centers for Disease Control and Prevention, Accra (D. Barradas)

**Keywords:** yellow fever, polio, COVID-19, vaccine deployment, Global Health Security Agenda, SARS-CoV-2, coronavirus disease, severe acute respiratory syndrome coronavirus 2, viruses, respiratory infections, zoonoses, pandemic, Ghana

## Abstract

Ghana is a yellow fever–endemic country and experienced a vaccine-derived polio outbreak in July 2019. A reactive polio vaccination campaign was conducted in September 2019 and preventive yellow fever campaign in November 2020. On March 12, 2020, Ghana confirmed its first COVID-19 cases. During February–August 2021, Ghana received 1,515,450 COVID-19 vaccines through the COVID-19 Vaccines Global Access initiative and other donor agencies. We describe how systems and infrastructure used for polio and yellow fever vaccine deployment and the lessons learned in those campaigns were used to deploy COVID-19 vaccines. During March–August 2021, a total of 1,424,008 vaccine doses were administered in Ghana. By using existing vaccination and health systems, officials in Ghana were able to deploy COVID-19 vaccines within a few months with <5% vaccine wastage and minimal additional resources despite the short shelf-life of vaccines received. These strategies were essential in saving lives in a resource-limited country.

Before and during the COVID-19 pandemic, Ghana experienced successes with vaccination campaigns for yellow fever and polio that influenced its deployment of COVID-19 vaccines (*1*,*2*). Ghana had successfully eliminated all 3 serotypes of wild polioviruses by 2008. In 2019, however, Ghana confirmed a case of circulating vaccine-derived poliovirus type 2 (cVDPV-2). As part of the response strategy, a reactive monovalent oral polio vaccine 2 (mOPV2) campaign targeting children <5 years of age was conducted in a phased approach across the country beginning in September 2019 (*1*).

Ghana is also among 34 yellow fever–endemic countries in Africa and experienced an outbreak of yellow fever in June 2014. As part of the efforts to prevent and control yellow fever, even during the COVID-19 pandemic, Ghana conducted a yellow fever preventive mass vaccination campaign in November 2020 among 81 districts in 14 (88%) of the 16 regions in the country. The campaign targeted ≈5.6 million persons 10–60 years of age and achieved administrative coverage of 94% in the targeted districts (*2*).

On January 30, 2020, the World Health Organization (WHO) declared COVID-19 a public health emergency of international concern, and on March 11, 2020, WHO declared COVID-19 a pandemic (*3*,*4*). In March 2020, Ghana confirmed its first 2 cases of COVID-19 (*5*). During February–August 2021, Ghana received 1,515,450 COVID-19 vaccines through the COVID-19 Vaccines Global Access initiative and other donors, such as the African Union, the Indian government, and United Arab Emirates (*6*). By March 14, 2020, when the first cases of COVID-19 were being identified, the country had achieved administrative coverage of 97% nationally for the mOPV2.

The COVID-19 response in Ghana has been coordinated by the Inter-Ministerial Coordination Committee (IMCC), which consists of cabinet members, key national government ministers and representatives from the President’s Office of Ghana, and key officers from health and other social service agencies. The IMCC is chaired by the president of Ghana. The IMCC identified COVID-19 vaccination as one of the key strategies for effectively responding to the COVID-19 pandemic and charged Ghana’s National Immunization Technical Advisory Group (NITAG) to develop a national COVID-19 vaccine deployment plan for Ghana.

Using proven microplanning and macroplanning strategies, national and subnational trainings were held to train supervisors, monitors, vaccinators, data collectors, and volunteers, and vaccines and other logistics were distributed to the 14 participating districts (*7*). In addition, supervisors were deployed to their respective field assignments. Phase 1 of the immunization campaign began within 7 days of vaccine receipt. We describe how the Expanded Program on Immunization (EPI) structures used for polio and yellow fever vaccine deployment and lessons learned from these previous campaigns were leveraged to quickly deploy COVID-19 vaccines in Ghana during March 1–August 23, 2021, regardless of the short shelf-life of vaccines received (1–3 months).

## Methods

### Setting

Ghana is a country in West Africa that shares borders with Togo, Cote d’Ivoire, and Burkina Faso. As of 2021, Ghana had a population of ≈30.08 million and an annual growth rate of 2.1% (*8*). The country provides free vaccination services. However, equity gaps remain a challenge among children >18 years of age of different socioeconomic backgrounds.

Ghana has 4 functional areas of the health system: administration and financing, healthcare service delivery, training, and regulatory. These functions are organized and implemented at the national, regional, and district levels, and some of these functions operate below the district level (i.e., subdistricts and health facilities). The Ghana Health Service (GHS), on behalf of the Ministry of Health (MoH), is the supervising agency for delivering primary and secondary healthcare services in the country, including immunization.

### Developing the COVID-19 National Vaccine Deployment Plan

A technical working group made up of implementing partners including the WHO, US Centers for Disease Control and Prevention (CDC), and departments and divisions under the MoH/GHS was constituted under the directive of the Minister of Health. The NITAG led plan development. The team was tasked to develop national guidelines that would serve as a road map for the deployment of COVID-19 vaccines in Ghana. The plan was developed under the guidance of the WHO Strategic Advisory Group of Experts on Immunization. The final decision on prioritization in Ghana was under the recommendation of the NITAG.

Data from the WHO COVID-19 weekly epidemiologic updates, polio and yellow fever surge officers’ reports, the District Health Information Management System, and the Surveillance Outbreak Response Management and Analysis System for COVID-19 Response were used to develop the Ghana COVID-19 Vaccine Deployment Plan. The plan described district prioritization on the basis of COVID-19 cases and deaths during March 2020–January 2021 (i.e., before vaccine deployment), enumerated priority groups (e.g., healthcare workers, persons >60 years of age, and persons with selected comorbidities, such as hypertension, diabetes, chronic lung disease, cancer, heart conditions, kidney disease, and other immunocompromising conditions) within districts, and a detailed near real-time assessment of districts’ readiness to receive, store, and distribute vaccines. The plan called for vaccines to be administered by using the population segmentation approach.

Vaccination teams collected real-time data using the Open Data Kit (https://getodk.org) on tablets and mobile phones; data were synchronized to a central database accessible at all levels of the health system. Contact details and geographic coordinates were also captured. Text message reminders were sent to those who were due for their second dose. If those persons did not respond, community health nurses and surveillance officers used information captured during the first vaccine administration to trace the person. Persons with comorbidities were identified through reviews of medical histories.

At the national and regional levels, a visual dashboard was used to monitor vaccination activities to provide near real-time updates to field officers. Cold chain management systems during the yellow fever and polio campaigns were also assessed to determine their current condition in terms of functionality and storage capacity and ascertain whether expansion was necessary to ensure equity across regions. The assessment of the cold chain management systems during the polio and yellow fever campaigns gave a snapshot of the cold chain capacity in Ghana before COVID-19 vaccine deployment.

During the polio and yellow fever campaign, field monitors were deployed across regional, district, and subdistrict levels to provide real-time feedback using the Open Data Kit tool on tablets and phones for immediate action. Lot quality assurance sampling surveys were conducted to ensure adequate coverage in hard-to-reach areas. Residents of the Ghana College of Physician and Surgeons conducted the surveys, and training was done by the Ghana Health Service and WHO.

Developing of plans for social mobilization, training, cold chain management, vaccine deployment, coverage tracking, and postimmunization safety monitoring relied on systems and personnel who had participated in the earlier polio and yellow fever vaccination campaigns. Before the yellow fever vaccination campaign, logistics support was needed for personal protective equipment and yellow fever vaccination cards. Within a period of 2 weeks, CDC Foundation raised enough funds to procure 87,841 (250-L) bottles of hand sanitizer worth US $224,371 and 5.6 million yellow fever vaccination cards worth US $130,000 to enable the yellow fever preventive mass vaccination campaign to proceed as planned.

In the area of regulatory preparedness and safety monitoring, the Ghana Health Service worked closely with Ghana Food and Drugs Authority (FDA) to assess the safety and efficacy of the yellow fever and polio vaccines before deployment. Because of the lack of capacity and resources to conduct clinical trials, Ghana FDA reviewed and validated reports from other stringent regulatory authorities. Other test parameters conducted by Ghana FDA included pH and UV tests. In addition, EPI and Ghana FDA collaborated to monitor adverse events after immunization, including causality assessment.

Lessons from these previous reactive campaigns were used to guide the proposed deployment strategies for COVID-19 vaccines. We calculated vaccine wastage rate by subtracting the total doses used from the total doses supplied, divided by the total dose supplied expressed as a percentage (*9*). We analyzed coverage and field supervision data for phase 1 of COVID-19 vaccine deployment by using Epi Info 7 (https://www.cdc.gov/epiinfo/index.html) and Power BI (https://powerbi.microsoft.com).

### Ethics Statement

This activity was part of the national pandemic preparedness response by the Ghana MoH and GHS and was not deemed human subjects research. As such, ethical clearance was not required (Act 851 Public Health Act, 2012, Ministry of Health, Ghana). Primary data generated were stored electronically on national servers and password protected. Only reports with no personal identifying information were shared with other stakeholders such as WHO, UNICEF, and CDC.

## Results

### COVID-19 National Vaccine Deployment Plan

The National Vaccine Deployment Plan (NVDP) in Ghana has 10 main components: planning and coordination; regulatory preparedness and safety monitoring; vaccination strategies; deployment systems and modalities; immunization monitoring system; operational research and surveillance; communication and information; supply chain processes; waste management; and monitoring and evaluation. The development of the components of the NVDP was based on lessons learned during the yellow fever and polio immunization campaigns (Table 1). On the basis of lessons learned from the resource mobilization for the yellow fever campaign, Ghana was able to mobilize both human and logistical resources from institutions and implementing partners such as WHO, USAID, CDC, and World Bank for COVID-19 vaccine deployment.

**Table 1:** Lessons learned during yellow fever and polio immunization campaign that informed the COVID-19 National Vaccine Deployment Plan, Ghana, September 4, 2019–November 18, 2020*

NVDP component	Lessons learned during yellow fever and polio immunization campaigns
Planning and coordination	Activate or establish an Incident Management System for coordinating the vaccination response
Regulatory preparedness and safety monitoring	Liaise early with the Ghana (or any country) FDA to ensure the evaluation and approval of vaccines before deployment and the monitoring of adverse events following immunization
Vaccination strategies	Develop detailed and accurate microplans inclusive of strategies for hard-to-reach areas.
Deployment systems and modalities	Identify resources early for surge deployment of human resources and logistics for vaccination activities and campaigns; deploy Field Epidemiology and Laboratory Training Program alumni and residents as surge staff
Immunization monitoring system	Establish sites and deploy field officers for safety monitoring and reporting
Operational research and surveillance	Conduct surveys to assess the knowledge, attitudes, practices, and behaviors of the target population toward vaccine acceptance at predefined time points
Communication and information	Use media scanning to understand the drivers of vaccine hesitancy and acceptance among various subpopulations. Use mass media, celebrities as ambassadors, and communication centers to increase vaccine demand and reduce hesitancy. For hard-to-reach areas, community leaders and opinion leaders were used to lead communications to address vaccine hesitancy
Supply chain processes	Improve cold chain capacity (a system to maintain a desired temperature for viability of vaccines in the supply chain) before there is an outbreak or epidemic; adopt and use a standardized process for vaccine accountability and retrieval
Waste management	Use incinerators to destroy waste generated by vaccination under supervision.
Monitoring and evaluation	Deploy field monitors at regional, district, and subdistrict level to provide real-time feedback, using the Open Data Kit for immediate action. Ensure that every vial is accounted for daily. After every campaign, a monitoring team consisting of Ghana FDA, Environmental Protection Agency, WHO, and UNICEF officials should oversee the incineration of empty vials by region and certify that all vials have been accounted for. The Vaccine Accountability Monitoring officers should undergo formal training to ensure accuracy

*FDA, Food and Drug Authority; NVDP, National Vaccine Deployment Plan; WHO, World Health Organization.

Findings from the assessment of the cold chain structures used for the yellow fever and polio vaccination campaigns were used to develop proposals to government and other donor agencies for funding support. As a result, 94 ultra-low freezers sponsored by the government of Ghana, the government of Japan, World Bank, and UNICEF were installed across the country.

In terms of communication and information, media scanning was used to understand the drivers of vaccine hesitancy and acceptance among various subpopulations. Historical approaches, such as using mass media, celebrity ambassadors, and communication centers to promote vaccine uptake and address hesitancy, were used for COVID-19 vaccine deployment. The president of Ghana and some members of his leadership team were vaccinated live on national television to promote vaccination. For hard-to-reach areas, community leaders and opinion leaders were used to lead communication efforts addressing vaccine hesitancy. Using lot quality assurance sampling surveys to ensure adequate coverage in hard-to-reach areas helped affirm the need to extend the campaign in those communities by an extra 3–7 days.

### Vaccine Deployment

As of August 23, 2021, a total of 1,424,008 doses of vaccines (1,282,097 AstraZeneca [https://www.astrazeneca.com], 15,813 Sputnik V [https://www.sputnikvaccine.com], and 126,178 Janssen/Johnson & Johnson [https://www.jnj.com]) had been administered in Ghana, with <5% vaccine wastage and minimal additional resources despite the short shelf-life of vaccines received (K. Amponsah-Achiano, GHS, unpub. data, 2021 Aug 23). At the end of phase 1, a total of 461,800 persons were fully vaccinated and ≈865,422 persons had received >1 dose. Deployment was based on population and geographic segmentation across the country. Most vaccinations occurred in the Greater Accra and Ashanti regions, the 2 epicenters of the COVID-19 epidemic in Ghana.

The initial phase of deployment during March 1–August 23, 2021, used a population segmentation approach that covered healthcare workers, frontline security personnel, persons with known comorbidities, teachers >50 years of age, persons >60 years of age, and frontline members of the executive, legislature, and judiciary sections of the government (Table 2; Figure). Of the 1,424,008 doses administered, >865,422 persons received their first dose, and 432,488 received their second dose. Overall, 558,666 persons were fully vaccinated (i.e., received 1 dose of Janssen/Johnson & Johnson vaccine or 2 doses of AstraZeneca or Sputnik V vaccine). About 52% of vaccine recipients were men (742,004) and 48% women (682,004).

**Table 2:** Performance of phase 1 COVID-19 vaccine deployment, Ghana, March 1–August 23, 2021

Phase	Start date	No. regions	No. districts	Target segmentation	No. doses administered
Phase 1A	2021 Mar 1	3	43	Most at-risk groups in 43 hotspot districts	535,408
Phase 1B	2021 Mar 24	13	217	All healthcare workers	316,639
Phase 1C	2021 May 19	3	43	All persons vaccinated in Phase 1A during March 1–9, 2021 (12 weeks after first dose)	380,829
Phase 1D	2021 Jun 20	16	260	2021 census enumerators (1st dose) and 2nd dose vaccinations in selected districts	65,034
Phase 1E	2021 Aug 13	3	11	General population in selected areas	126,178
Total					1,424,088

**Figure:**
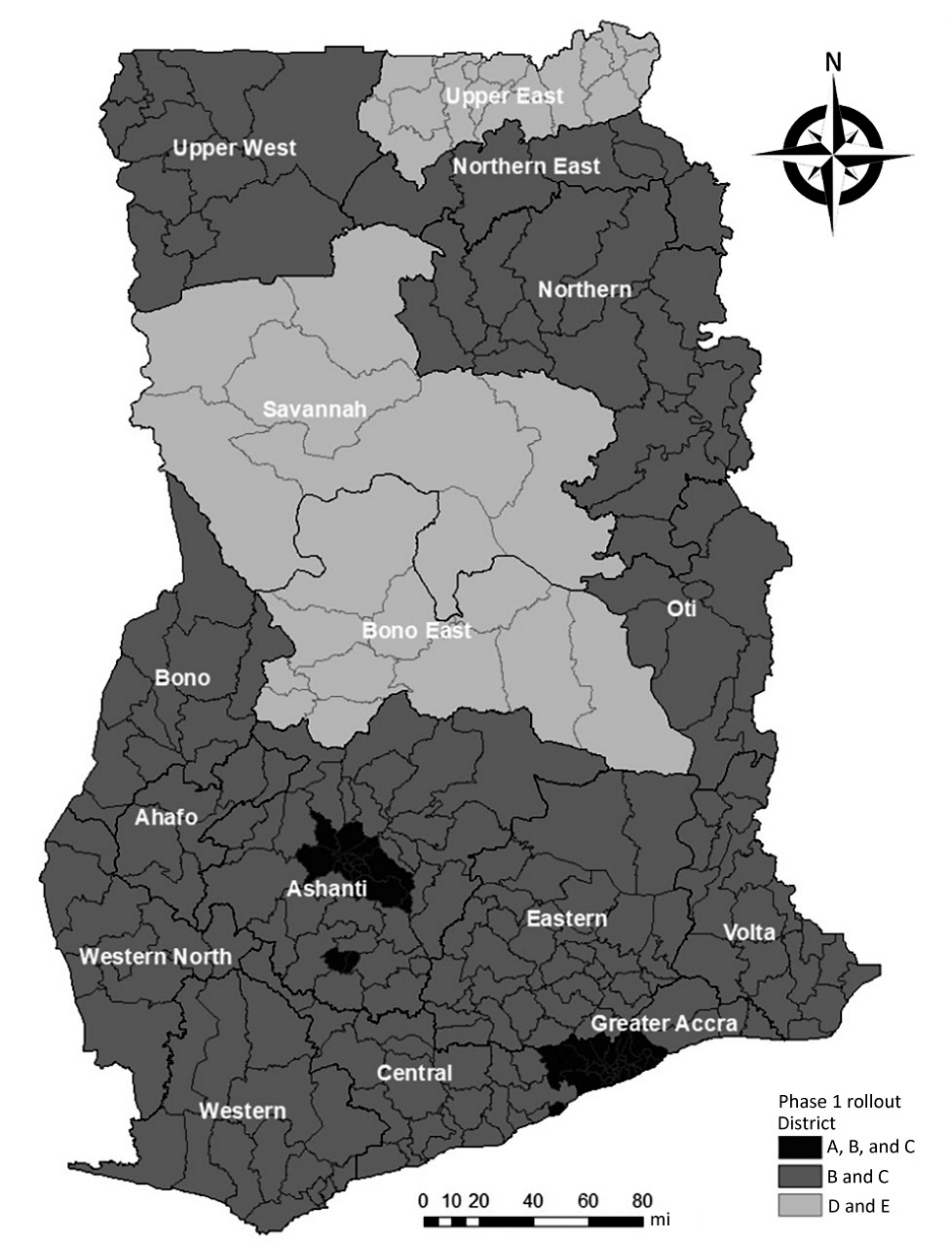
Distribution of phase 1 COVID-19 vaccination campaign deployment by district, Ghana, March 1–August 23, 2021. Based on population and geographic segmentation, phase 1A targeted the most at-risk groups in 43 hotspot districts; phase 1B targeted all healthcare workers; phase 1C targeted all persons vaccinated in phase 1A during within March 1–9, 2021 (12 weeks after first dose); phase 1D targeted 2021 census enumerators (first dose) and second-dose vaccinations in selected districts; and phase 1E targeted the general population in same districts. Phase 1E vaccination was with the Janssen/Johnson & Johnson vaccine (https://www.jnj.com).

## Discussion

We describe how Ghana leveraged existing vaccination programs and structures and lessons learned from previous vaccination campaigns to ensure effective and efficient deployment of COVID-19 vaccines. The Ghana Technical Working Group for COVID-19 vaccine deployment ensured a comprehensive approach to COVID-19 deployment in Ghana.

The population segmentation used in administering the vaccines ensured that the most at-risk groups were covered to reduce severity of the disease. This approach has also been used in India (*10*). An incident management system for COVID-19 vaccine deployment established within the National Public Health Emergency Operations Center served as a platform for resource mobilization and reduced duplication among stakeholders and partners (*11*–*13*).

Regarding regulatory preparedness and safety monitoring, one of the lessons learned from the yellow fever and polio campaigns was the need to liaise early with the Ghana FDA to ensure the evaluation and approval of vaccines before deployment. All vaccines need Ghana FDA approval before they can be administered in the country. The close collaboration between Ghana FDA and EPI helped ensure a plan for monitoring adverse events after immunization to avoid duplicating efforts and harness data sharing for decision-making.

During the yellow fever and polio vaccination campaigns, rural communities in hard-to-reach areas representing ≈10% of the target population were not vaccinated because the communities were geographically remote relative to other campaign areas and could not be covered within the days allocated. To address this problem, the vaccination periods during the COVID-19 campaign were extended for a week in those locations. Separate campaigns were organized for communities that were cut off because of flooding and required special arrangements, such as use of canoes. All these considerations were factored into the COVID-19 vaccine deployment microplans. Capitalizing on existing microplanning strategies helped reduce the time for vaccine deployment for COVID-19 by ≈50% and ensured equity in vaccine distribution to rural, urban, and hard-to-reach communities within earmarked regions and districts.

One of the strategies that led to efficient deployment systems and modalities was early planning and identifying resources for surge deployment of human resources and logistics for vaccination activities and campaigns. This approach involved engaging institutions such as the Field Epidemiology and Laboratory Training Program to deploy alumni and residents as surge staff (*14*). Deploying surge officers during the yellow fever and polio vaccination campaigns in Ghana and Nigeria improved vaccination by increasing the number of routine immunization outreach sessions and expanding vaccine coverage, conducting active case searches and social mobilization, and strengthening partnerships with key stakeholders (*15*). This same strategy was used for the COVID-19 vaccination campaigns. Implementing partners also deployed field officers to support the monitoring activities.

Cold chain management is known to be an essential component of vaccine deployment, as has been reported in countries such as Tunisia (*16*,*17*). The cold chain in Ghana was revamped with support from Gavi, the Vaccine Alliance. The use of lot quality assurance sampling surveys in ensuring adequate coverage in hard-to-reach areas was demonstrated during previous vaccination campaigns in Ghana (*18*,*19*). The strategy of using celebrities, media houses, and social media influencers for social mobilization has also proven to be an effective approach for social mobilization in Ghana and other countries such as Hong Kong, France, and the United Kingdom (*20*–*22*).

Other lessons were also learned from the COVID-19 vaccine deployment in Ghana. We identified the need to further expand cold chain capacity to enable the storage of larger quantities of vaccines, because the country’s previous capacity for vaccine storage was limited. In addition, ultra-cold chain capacity needed to be decentralized because capacity was only at the central and regional level and some selected facilities. This decentralization would require additional resources to transport vaccines to other parts of the country while maintaining the cold chain. With respect to leadership, we learned that successful vaccine deployment thrives on commitment by the country’s leadership, which was evident at all levels of the health system. However, a limitation of our study is that the lessons learned from the yellow fever and polio campaign were drawn from 81 districts and might not be representative of the entire country.

Using existing systems to deploy COVID-19 vaccines saved time and resources. Ghana was able to deploy its COVID-19 vaccines within a short time (3 weeks–2 months after receipt of vaccines), which was essential because of the short shelf-life (1–3 months) of the vaccines. Unlike many countries that were not able to use all received vaccines before expiration, Ghana had <5% vaccine wastage. The country is currently planning for subsequent phases of vaccine deployment and providing technical assistance to other neighboring countries, such as Cote d’Ivoire, on strategies for effectively deploying COVID-19 vaccines.
